# Policy brief of the Belgian Europe’s Beating Cancer Plan mirror group: children, adolescents and young adults with cancer

**DOI:** 10.1186/s13690-024-01366-6

**Published:** 2024-08-27

**Authors:** Hélène A. Poirel, Gabrielle Schittecatte, Fabienne Van Aelst, on behalf of the Thematic Working Group (TWG) ‘Children, Adolescents and Young Adults (CAYAs) with Cancer’, Ad Vandermeulen, Alice Gerbaux, Christiani Andrade Amorim, An Van Damme, Anne Uyttebroeck, Benedicte Brichard, Bram De Wilde, Brecht Gunst, Caroline Stals, Chloë De Witte, Christiane Jungels, Christine Devalck, Claire Geurten, Daan Dierickx, Delfien Bogaert, Delphine Heenen, Elien Romaen, Elisa Balducci, Els Van Valckenborgh, Evi Lippens, Franck Dequiedt, Gabriel Levy, Hans Neefs, Heidi Segers, Ibrahim Chiairi, Isabelle Demeestere, Johan De Munter, Joris Verlooy, Julie Messiaen, Jurgen Lemiere, Karen Van Beek, Karen Vandenabeele, Katrien Maes, Kleo Dubois, Laura Polastro, Laurens Van Camp, Lien Van De Voorde, Lore Lapeire, Manon Le Roux, Marcela Chavez, Margherita Condorelli, Marie Caillier, Marie Vander Haegen, Nancy Van Damme, Nathalie Belpame, Olga Kholmanskikh, Paulina Bartoszk, Pauline Mazilier, Pierre Mayeur, Pierre Philippet, Saar Vandekeere, Sabine Verschueren, Sandra Jacobs, Sofie Moreels, Stefan Gijssels, Tom Boterberg, Toon Van Genechten, Valentina Albarani

**Affiliations:** Sciensano, Cancer Centre, Sciensano, Brussels, Belgium

**Keywords:** Childhood cancer, Adolescents and Young Adults (AYA) with cancer, Europe’s Beating Cancer Plan (EBCP), Policy Brief, Policy Recommendations

## Abstract

Children and Adolescents and Young Adults with cancer represent a young population with specific needs, which need to be addressed in a patient- and cancer-driven way. There is an urgent need to support and extend the ongoing initiatives in Belgium. First, multidisciplinary care programmes dedicated to children need to be reviewed, and those for Adolescents and Young Adults need to be developed with close collaboration between paediatric and adult oncology and haematology teams. This needs to be done considering the entire patient journey; from cancer prevention, diagnosis, treatment, rehabilitation, follow-up of late effects, transition pathways between paediatric and adult wards, and palliative care. Second, national haemato/oncology precision programmes adapted to this young population with rare cancers, including infrastructure to manage cancer gene predisposition in CAYAs with cancers and their relatives, needs to be developed. This multi-level plan aims to ensure improved outcome with high quality of care for the young population with cancer in Belgium in line with Europe’s Beating Cancer Plan initiatives.

## Background

Europe’s Beating Cancer Plan (EBCP) is putting childhood cancer under the spotlight [1] to ensure that children have access to rapid and optimal care. It also seeks to support training of healthcare professionals, sharing of best practices, complementing the actions implemented by the European Reference Network on Paediatric cancers (ERN PAEDCAN [2]). Other initiatives also focus on the young population with cancer, such as the childhood cancers and cancers in adolescents and young adults (AYA) initiative to “boost the transformation of paediatric cancer care” [1] and to establish an EU Network of Youth Cancer Survivors (EU-CAYAS-NET [3]). These European initiatives are a great present and opportunity to tackle cancers in Belgian children and AYAs.

AYAs with cancer represent a distinct and heterogeneous population interposed between paediatric and adult oncology/haematology, with modest survival gains and challenging unmet needs. Internationally, there is no clear consensus about the age limits (e.g., from 13–18 to19-39 year-old) in this group [4]. Recent joint recommendations from the European Society for Medical Oncology (ESMO) and the European Society for Paediatric Oncology (SIOPE) emphasize the importance of the close collaboration between paediatric and adult teams, and of the patient- and cancer-driven care (rather than age-driven), without any predefined organizational model [4].

The Thematic Working Group (TWG) of the Belgian EBCP mirror group on ‘Paediatric cancers’ initially discussed issues and Belgium’s participation in European Union calls for children and adolescents. In 2023, this TWG was extended to AYAs with cancers to address their specific needs and renamed as TWG on CAYAs with cancer.

This policy brief, prepared through the discussions of the TWG, outlines the major initiatives related to children and preliminarily to AYAs with cancer in Belgium and proposes key policy recommendations to fill the gaps regarding the specific needs of this target population in order to provide high quality care.

## Main text

### Belgian context

In 2021, 316 children (0–15 year-old), 107 adolescents (16–19) and 1691 young adults (20–35) in Belgium were diagnosed with a malignant neoplasm (excluding non-melanoma skin cancer). The 10-year relative survival was 83% for children (3073 N at risk) and 87% for AYAs (16–35) (17,076 N at risk) diagnosed between 2012 and 2021 [5].

Paediatric haemato/oncology (PHO) consists of more than 60 rare malignancies, treated exclusively in seven Belgian PHO centres, according to the Royal Decree of 2014 (RD) [6]. The standard of care is the inclusion in a multinational academic study, when possible, and maximised participation in early phase studies. The RD (currently under revision) sets the criteria that a PHO care programme must meet to be approved [6]. Programmes should cover diagnosis, multidisciplinary treatment, rehabilitation, follow-up of late effects and palliative care for all patients under 16 years. The Belgian Society of PHO (BSPHO) strongly supports the scientific collaboration between the 7 PHO centres (including the set-up of clinical trials).

Young people aged 16 and over who are diagnosed with cancer are referred to the adult haemato/oncology department in Belgium, with some flexibility for adolescents to decide whether they prefer to be treated in paediatric or adult ward. For the period 2018–2021, a yearly average of ≥ 50, 20–49, 5–19 and 1–4 AYAs with cancer to the Belgian Cancer Registry (BCR) were respectively registered in 6, 26, 48 and 20 Belgian hospitals [5]. A recent policy note from the health authorities underlines the concern for the young population, in terms of quality of care, accessibility as well as the development and the sharing of knowledge [7].

### Projects, Gaps

#### Cancer care

##### Children

Medical and paramedical staffing norms for PHO in Belgium remain one of the lowest in Europe. Human resources are very limited in Belgian PHO centres, compared to similar centres in other European countries, making it difficult to maintain qualitative standards of care, given the increasing complexity of diagnostic and therapeutic developments.

Since 01/01/2024, the National Institute for Health and Disability Insurance (RIZIV/INAMI) allocated a yearly budget of 3.2 Mio € for the reimbursement of 52 chemotherapy and supportive care drugs used ‘off-label’, which are part of the standard of care [8]. As stated above, qualitative standard of care of children with cancer involves inclusion in clinical trials, given the rarity of all paediatric cancers. This means that an important part of first-line management of children with cancer is financed through research grants, mostly funded by philanthropic organisations.

##### Adolescents and young adults

There is an urgent need to organise the care of AYAs with cancer in Belgium and to develop and disseminate the expertise in such care, with the ultimate aim of improving outcomes and quality of life. A national program dedicated to AYAs with cancer started at the end of 2023, with resources allocated for AYA-multidisciplinary teams in 6 hospitals with recognized expertise in both adult and paediatric oncology/haematology, and which treat more than 50 AYAs (16–35) with cancer yearly [9]. The convention between these hospitals and RIZIV/INAMI aims to provide tailored medical and psychosocial care, and to share expertise with other hospitals and first line care. This is organised within a project group, together with AYA-experts and patient organisations, which is coordinated by Sciensano. This initiative relies on pre-existing projects, such as the Blueprint for AYA-care in Flanders [10], Care4AYA [11], tailor-made trainings for healthcare professionals delivered by the Cédric Hèle Instituut [12], and fertility preservation program with multicentric prospective trial conducted by the University Hospital of Brussels (HUB). They also benefit and feed JANE, the European project which aims at developing a network of expertise on AYAs ([13]).

##### Integration of innovations into care

There is a clear gap in adoption of innovations for paediatric and AYA-cancers in Belgium. The TEARDRoP-consortium is being set up in Belgium, under the umbrella of the BSPHO, to bridge the gap between basic and clinical research to identify new therapeutic options for children with cancer, through supporting collaborations and biobanking between the different PHO-centres [14].

Available targeted gene tests in Belgium need to be supplemented by a more comprehensive molecular profiling (CMP) (whole genome, transcriptome and epigenome sequencing) for selected cases, for which no standardized therapeutic strategy exists. As proof-of-concept studies have resulted in patient benefit, other leading European countries are already incorporating these advanced CMP-programs into their routines. A limited number of children and adolescents in Belgium have benefited from a CMP carried out abroad and funded by charities via the BSPHO. These initiatives need support and extension in the framework of a CAYA-haemato/oncology precision program.

##### Oncogenetics

The burden of genetic susceptibility in the young population is underestimated (~ 10% of CAYAs in large-scale sequencing studies). Infrastructure is currently being developed by UZGent for whole exome sequencing with genetic counselling in young patients with suspicion of genetic cancer predisposition. It aims to be multicentric, with potential extension to AYAs, owing to the participation in European projects Can.Heal [15], and JAPreventNCD [16]. These pilot projects need to be further supported and expanded to prevent and/or early detect cancer in predisposed CAYAs and their relatives.

##### Cancer survivorship

Several pilot projects are underway in Belgium in the paediatric setting that needs to become sustainable and extended beyond 18 years. The registration project *Paediatrics—Late effects* [17] seeks to gain more insight into acute and late side effects in children and adolescents (0–19) treated for cancer. This registration project is conducted in collaboration with the BCR, under the umbrella of BSPHO and funded by charities. Fertility issues will be analysed in 2024, partly using data from PRINCESS [18]. Additionally, the international multicentric prospective trial *CHANCE* aims to evaluate survivors’ fertility predictors through long-term ovarian function assessment of pre-pubertal and pubertal children treated by chemotherapy [19]. The goal of the pilot study conducted at the University Hospital of Leuven in the context of the *EU PanCareSurPass Project* [20] is to prepare the challenging implementation of a digital survivorship passport to improve people-centred care for childhood cancer survivors. Finally, Belgian expertise was recognized via their participation as experts in the recent international guidelines on fertility preservation in children and AYA [21, 22].

Nonetheless, clinical activities regarding long-term follow-up of survivors and their transition to adult follow-up require specific expertise and dedicated time of physicians of multiple specialities, which currently is not recognized as such in Belgium, and not reimbursed (preventive medicine).

##### Palliative care

The Belgian Paediatric Palliative Care (BPPC) [23] expert group aims to develop a plan to broaden the scope and increase awareness of paediatric palliative care. Guidelines have been established.

However, the scientific secretariat provided by the Cancer Centre (Sciensano) [24] has not been funded since 2022. Moreover, palliative care dedicated to AYAs with cancer is lacking in Belgium.

### Policy recommendations

#### Care organisation

Multidisciplinary care programmes for paediatric cancers need to be reviewed (e.g., criteria for recognition of paediatric treatment sites and teams, adaptation of human resources). Multidisciplinary care programmes dedicated to AYAs need to be developed and recognized in Belgium. The core goal should be the creation of dedicated units in recognized expert AYA-hospitals. The RIZIV/INAMI-convention financing AYA-reference teams in 6 hospitals (ref. Section 2.1.2) is a first step in establishing dedicated AYA-centres and programs.

The weekly national cancer board organised by the BSPHO for children need sustainable financial support and incentives to ensure the participation of the relevant specialists (e.g., surgeons, radiation oncologists, geneticists). A similar initiative should be developed for AYAs in the expert centres with close collaboration between paediatric and adult teams.

Patients on the frontier of paediatric and AYA-age groups suffer from lack of reimbursement of standard treatments (e.g., drugs, innovative radiotherapy techniques) due to arbitrary age limits in reimbursement criteria. We recommend revision and adaptation of these criteria to make them meet the current scientific standards.

#### Integration of innovations into care

There is an urgent need to make precision haemato/oncology more accessible to the CAYA-population, as for older adults, even if it represents a small number of cases and rare cancers. Comprehensive molecular profiling should be reimbursed in recognized indications for CAYAs.

We recommend:

- Support of the organisation of a national molecular board should be directly linked to current initiatives developed for adults as well as connected with international initiatives (e.g., ERN PAEDCAN, the future network of expertise on AYAs);
- Access to treatments for CAYAs remains a priority. Regulations and governance frameworks must be addressed to improve access to early phase clinical trials, in addition to the systematic reimbursement of standard of care off-label drugs [25]. Such access to (inter)national clinical trials may be improved by a network between existing clinical trial units that considers AYA-specific gaps and needs. Academic clinical trials in first-line treatment for CAYAs need to be funded. These could take the form of grants/subsidies and could benefit from the BSPHO experience with its centralised National Coordination Cell;
- There is a need to develop specific paediatric and AYA-cancer research programs in collaboration with international initiatives as well as to better use demand-driven policy instruments to overcome market failures.

#### Oncogenetics

We recommend developing the infrastructure and pipe-line to manage genetic cancer predisposition in CAYAs with cancers and their relatives:

- Belgian network of expertise with trained genetic counsellors;
- Genomic platform with reimbursed genomic screening for genetic cancer predisposition in all children and in selected AYAs, as part of genetic counselling;
- Appropriate long-term surveillance programmes for CAYAs with cancer and their relatives with genetic predisposition, including psycho-social follow-up and potential reproductive implications;
- Decision support tools for practitioners connected to updated guidelines and a national database.

#### Follow-up after treatment and transition paths

A specific reimbursement for multi-disciplinary long-term follow-up consultations with recognition of all involved healthcare professionals should be put into place, so that a more structured transition path between paediatric and adult haemato/oncology can be ascertained, as already established in other European countries. This will benefit from the implementation of the electronic Survivorship Passport for each child and AYA who has had cancer, with an extension of this current project beyond 18 years.

We recommend the recognition of revalidation programs for children and AYAs patients with prospective registration and financial protection through insurance. It is also important to support BPPC and develop AYA-palliative care.

## Conclusion

This multi-level plan aims to ensure improved outcome with high quality of care for the young population with cancers in Belgium, by developing comprehensive infrastructures and networked expertise adapted to the specific needs of these patients. This is also expected to benefit rare cancers in older adults. Long-term follow-up and a structured transition path will improve the (re)integration of CAYAs into society and ensure their future quality of life. As for the other policy briefs reported in this supplement, we hope that this one focusing on CAYAs with cancer will help policy-makers in Belgium to take decisions and contribute to the ongoing European initiatives.

## Data Availability

Not applicable.

## Abbreviations

AYAs: Adolescents and Young Adults
BCR: Belgian Cancer Registry
BPPC: Belgian Paediatric Palliative Care
BSPHO: Belgian Society of Paediatric Haematology Oncology
CAYAs: Children, Adolescents and Young Adults
CMP: Comprehensive Molecular Profiling
EBCP: Europe’s Beating Cancer Plan
ERN PAEDCAN: European Reference Network on Paediatric Cancers
ESMO: European Society for Medical Oncology
EU-CAYAS-NET: EU Network of Youth Cancer Survivors
HUB: University Hospital of Brussels
INAMI-RIZIV: National Institute for Health and Disability Insurance in Belgium
JANE: Joint Action on Network of Expertise
JAPreventNCD: Joint Action on Prevention of Non Communicable Diseases
PHO: Paediatric Haemato/Oncology
PRINCESS: PReserving fertility and quality of life IN Belgian female paediatric CancEr SurvivorS
RD: Royal Decree
SIOPE: European Society of Paediatric Oncology
TEARDRoP: Team EARly DRug development in Pediatric oncology
TWG: Thematic Working Group
